# The degree of serum estradiol decline in early and midluteal phase had no adverse effect on IVF/ICSI outcome

**DOI:** 10.4103/0974-1208.63118

**Published:** 2010

**Authors:** Sachin A Narvekar, Neelima Gupta, Nivedita Shetty, Anu Kottur, MS Srinivas, Kamini A Rao

**Affiliations:** Department of Reproductive Medicine, Bangalore Assisted Conception Center, #6/7 Kumara Krupa, High Grounds, Bangalore - 560 001, India

**Keywords:** Clinical pregnancy, IVF/ICSI-ET, luteal phase, estradiol decline, ongoing pregnancy, preclinical abortion

## Abstract

**BACKGROUND:**

Estradiol levels fall rapidly in the luteal phase of ART cycles. So far, the effect of this estradiol decline on pregnancy outcome has remained controversial.

**AIM:**

To study the effect of early and midluteal estradiol decline on pregnancy and miscarriage rate. We also sought to determine whether estradiol fall was related to increased risk of bleeding per vagina in the first trimester among pregnancies which crossed 12 weeks.

**SETTING:**

Tertiary Assisted conception center.

**DESIGN:**

Retrospective study.

**MATERIALS AND METHODS:**

We analyzed data of 360 consecutive patients who underwent IVF-ET/ICSI cycles using one of the three protocols: Midluteal downregulation, short flare, and antagonist protocol.

**STATISTICAL METHODS:**

Statistical evaluation was performed with the Student's *t* test, Chi square, Fischer's exact test, analysis of variance, and Mann-Whitney tests were appropriate using SPSS for Windows, Standard version 11.0.

**RESULTS:**

The mean % EL-E2 and % ML-E2 declines were not significantly different in the pregnant and nonpregnant groups when analyzed separately in the three protocols. Also, the degree of midluteal estradiol decline did not correlate with pregnancy outcome. Moreover, the mean % early and midluteal estradiol decline did not differ significantly in patients with preclinical, clinical abortions, and ongoing pregnancy. The estradiol decline was not found to influence the risk of bleeding in the first trimester.

**CONCLUSIONS:**

Our results show that the degree of estradiol fall in the luteal phase of ART cycles does not influence pregnancy and first trimester miscarriage rate.

## INTRODUCTION

The synchronized effect of estrogen followed by progesterone is necessary to produce a receptive endometrium, which is essential for blastocyst implantation. In the follicular phase, it is well known that the action of estrogen causes proliferation of surface epithelium, glands, stroma, and blood vessels in endometrium and upregulation of progesterone receptors. In the luteal phase, however, the role of estradiol is not clear.

In assisted reproduction (ART) cycles, the use of GnRH agonists and antagonists to suppress the premature LH surge, along with the massive doses of gonadotropins for ovarian stimulation, creates a unique endocrine milieu in the luteal phase. The luteal phase in these cycles has been noted to be truncated with the duration of ovarian steroid production usually shorter by 1-3 days due to an abrupt decline in serum estradiol (E2) and progesterone (P4) levels, which occur after ovum pickup (OPU).

It is well established that the iatrogenic corpus luteal deficiency necessitates P4 supplementation in the luteal phase. However, there is no agreement on whether the accompanying E2 fall is detrimental to pregnancy outcome, and if so, whether luteal E2 supplementation would improve the outcome.

Most researchers investigating the effect of estradiol decline in the luteal phase of ART cycles have studied GnRh agoinst downregulation protocols, with little emphasis on the antagonist protocols. Secondly, major emphasis has been given to midluteal E2 decline. It would be important to know the effect of E2 decline earlier on, rather than in the midluteal phase, when the embryo implantation is expected to have already occurred, so as to ensure timely E2 supplementation.

In this retrospective study, we sought to determine the impact of early and midluteal E2 fall on pregnancy outcome, in the three commonly used COH protocols: Midluteal downregulation, short flare, and antagonist protocols. In order to determine whether the estradiol fall would disrupt the endometrial integrity, as proposed by some authors, we studied the impact on miscarriage rate and the risk of bleeding per vaginuum in the first trimester.

## MATERIALS AND METHODS

### Study design and subject selection

This is a retrospective study involving consecutive women undergoing IVF-ET treatment at our Institute, from the period between November 2006 and February 2008. Due to the nature of this study, no approval was obtained from the Institutional Review Board.

Inclusion criteria: Women aged less than 38 years undergoing fresh autologous IVF/ICSI-ET cycles were included. Only the first treatment cycle was analyzed for individual patient.

Exclusion criteria: Age >38 years, cycles in which embryo transfer (ET) was not done.

### Treatment protocol

According to our internal protocol, all patients were evaluated with baseline day 3 FSH, antral follicle count, and a hysteroscopy on 7^th^ to 10^th^ day of the cycle prior to the ET cycle. Controlled ovarian hyperstimulation (COH) was performed using one of the three protocols the long midluteal phase GnRH agonist suppression, GnRH antagonist, or the GnRH agonist short protocol as determined by their primary physician depending on age, antral follicle count, serum FSH levels, and response to previous ovarian stimulation protocols used for intrauterine inseminations.

In the long protocol, patients were downregulated with 0.5 mg of GnRH (Leupride® Sun Pharmaceuticals, Halol, Gujarat, India Ltd.) for a period of 10-14 days following which the dose was reduced to 0.2 mg and continued till hCG. After confirming adequate downregulation, FSH (Recagon® Organon, Ireland Ltd.), in the dose ranging from 150 to 250 IU was commenced depending upon the response of the previous cycle.

In the antagonist group, flexible, multiple-dose regimens were used. GnRH antagonist (Orgalutran® Organon Ireland Ltd.) was started at a dose of 0.25 mg when at least one follicle reached the size 14 mm. Both Recagon® and Orgalutran® were continued till ovulation trigger.

The patients allocated to the short protocol were administered GnRH agonist 0.5 mg from day 2 onward and continued till ovulation trigger. Gonadotropins were started from day 3 onward. Subsequent monitoring was same as in the long protocol.

Women were scheduled for oocyte retrieval when at least three follicles reached a size of 18 mm. Oocyte retrieval was performed by the transvaginal route under ultrasound guidance, 35-hr after HCG trigger, with the patient under conscious sedation. The morphology of each aspirated oocyte was noted after denudation with hyaluronidase.

The embryos were classified as follows:

Grade 1 - preembryos with blastomeres of equal size and no cytoplasmic fragmentation;

Grade 2 - preembryos with blastomeres of equal size with cytoplasmic fragmentation equal to 15% of the total embryonic volume;

Grade 3 - uneven blastomeres with no fragmentation;

Grade 4 - uneven blastomeres with gross fragmentation (≥20% fragments).

Veeck[1] grades 1 or 2 embryos were considered to be good- quality embryos. ICSI was performed for severe oligo- anthenozoospermia. Combined (conventional IVF and ICSI) were used in some patients with unexplained infertility. ET was performed with a Wallace® catheter (Smith Medical International Ltd., Hythe, Kent, UK) on days 3, 4, or 5 atraumatically under ultrasound guidance by a senior consultant.

Assisted hatching was not done in any of the patients. Luteal phase was supported with 600 mg/day of micronized progesterone vaginally till 12 weeks of pregnancy. In addition to this, midluteal downregulated cycles received 5000 IU of HCG on days 5 and 9 after ET provided the peak E2 was < 4000 pg/ml.

Venous blood was obtained to determine the E2 levels in the morning of HCG trigger, 8 hr after oocyte retrieval and in the morning 1 week after ET.

Assays of E2 were performed using Advia Centaur CP competitive immunoassay system (Siemens Medical Diagnostics, Germany).The minimum detection limit was 70 p mole/ml. The intra- and interassay coefficients of variation were 4.3% and 5.5% for low control and 5.1% and 7% for high control.

*P* values < 0.05 were considered significant.

The drop in serum E2 level was calculated as follows:

*Early E2 decline (EL*-*E2*) - ratio of the E2 on day of OPU (early-E2) to E2 on day of hCG (hCG-E2)

*Midluteal phase E2 decline (ML*-*E2*) - ratio of the E2 seven days after ET (ML-E2) E2 on day of hCG (hCG-E2)

The ratios of E2 thus obtained were used to calculate the %E2 decline from the formula 100-(E2 ratio × 100) and expressed as %EL-E2 fall and %ML-E2 fall.

### In our study, we used the following definitions

*Clinical pregnancy* - pregnancy with cardiac activity on transvaginal sonography

*Preclinical abortions* - patients who had abortion following biochemical pregnancy

*Clinical abortions* - patients who had miscarriage after the appearance of cardiac activity

*Ongoing pregnancy* - patients who crossed 12 weeks of gestation

### Statistical analysis

Statistical evaluation was performed with the Student's *t*-test, Chi square, Fischer's exact test, and analysis of variance (ANOVA), Mann-Whitney tests where appropriate using SPSS for Windows, Standard version 11.0. Differences were considered significant at *P* < 0.05. The parameters percent early and midluteal E2 decline in conception and nonconception cycles were evaluated by receiver operating characteristics analysis and calculation of the area under the curve to estimate their ability to discriminate between the two outcomes.

## RESULTS

We analyzed the baseline characteristics, hormonal profile, and the IVF outcome of 360 consecutive patients who met the inclusion criteria. The data was retrieved from our computerized database and verified by checking the individual case chart. Overall 146 patients conceived of which 24 had preclinical abortion, 21 had first-trimester clinical abortion, and from the 99 ongoing pregnancies 87 resulted in live birth. Of the 21 clinical abortions, 13 were missed abortions and rest resulted in spontaneous miscarriages. However, chromosomal analysis was not done for any of the products of conception. There were 120 patients in the clinically pregnancy group and 214 in the nonpregnant group. The mean ages in the two groups were 31 ± 4.2 and 31.8 ± 4.1, which did not differ significantly. The two groups were comparable in the day 3 FSH, indication for treatment (data not shown), mode of insemination, and amount of gonadotropins. The number of clinically pregnant women was significantly more in those undergoing midluteal downregulation. There were significant differences in the number of preovulatory oocytes retrieved and number of cleaved embryo's and the endometrial thickness on the day of ET, in the two groups [Table 1].

**Table 1:** Baseline characteristics

Characteristics	Clinical pregnancy (*N* = 120)	Nonpregnant (*N* = 214)	*P* value
Age	31 ± 4.2	31.8 ± 4.1	0.14
Day 3 FSH	5.3 ± 1.6	5.7 ± 2	0.13
Long protocol (%)	84 (70)	115 (53.7)	0.009
Short protocol (%)	10 (8.3)	19 (8.9)	
Antagonist protocol (%)	26 (21.7)	80 (37.4)	
Mode of insemination (%)			
IVF	76 (63.3)	110 (51.4)	0.1
ICSI	40 (33.3)	96 (44.9)	
Combined (IVF + ICSI)	4 (3.3)	8 (3.7)	
Amount of recombinant	1462.7 ± 3.89	1480 ± 423.4	0.7
FSH (IU)			
No. of preovulatory	6.9	5.3	0.000
oocytes			
No of oocytes	12.1 ± 6.6	9.9 ± 6.3	.003
No. of cleaved embryos	5.9 ± 3.1	4.65 ± 2.7	0.000
No. of embryos transferred	3.49 ± 6.06	3.18 ± 0.06	.06
Day of Embryo transfer (%)			
Day 2	7 (5.8)	24 (11.2)	0.3
Day 3	111(92.5)	187 (87.4)	
Day 4	2 (1.7)	2 (0.9)	
Day 5	-	1 (0.5)	
Peak E2 (pg/ml)	2407.8 ± 1376	2305 ± 157	0.5
Peak P4 (nmol/l)	3.16 ± 2.3	3.3 ± 2.1	0.4
Day 7 E2 (pg/ml)	630 ± 576	699 ± 759	0.3

### Hormonal profile

The absolute value of peak E2, peak progesterone, day 7 E2, and day 7 progesterone did not differ significantly [Table 1]. The mean % EL-E2 and % ML-E2 declines were not significantly different in the pregnant and nonpregnant groups when analyzed separately in the three protocols [Table 2].

**Table 2:** Impact of early and midluteal E2 fall on pregnancy outcome when compared in the three protocols

Protocol	Clinical pregnancy	Not pregnant	*P* value
Midluteal down			
Regulation			
%EL-E2 decline	39.6 ± 18.6	38.6 ± 20.99	0.7
%ML-E2 decline	74.2 ± 25.8	75.6 ± 26.7	0.7
Short flare			
%EL-E2 decline	40.2 ± 24.3	39.4 ± 17.4	0.9
%ML-E2 decline	50.1 ± 33	68.5 ± 26	0.2
Antagonist			
%EL-E2 decline	37.7 ± 25.1	37.2 ± 21.5	0.9
%ML-E2 decline	65.9 ± 25.6	61.6 ± 29.3	0.5

The degree of % ML-E2 fall when subcategorized into four groups (<25%, 25-49%, 50-74%, and >75% decline) did not have an impact on pregnancy rate [Table 3].

**Table 3:** Impact of degree of midluteal E2 decline on pregnancy outcome

% ML-E2Decline	Clinical pregnancy (%) (N = 120)	Nonpregnant (%) (N = 214)	*P* value
<25	19 (15.8)	47 (22)	0.4
25-49	17 (14.2)	34 (15.9)	
50-74	19(15.8)	32 (15)	
>75	65 (54.2)	101 (47.2)	

Moreover, we did not find significant difference in %E2 decline (early and midluteal E2) and the preclinical and clinical abortions and ongoing pregnancy [Table 4].

**Table 4:** Impact of Early and midluteal E2 fall on first trimester miscarriage

E2 decline (%)	Preclinical abortion (N = 24)	Clinical abortion (N = 21)	Ongoing pregnancy (N = 99)	*P* value
EL-E2 decline	36 ± 19	37.4 ± 25	39.3 ± 19	0.8
ML-E2 decline	68.5 ± 29	72.4 ± 30.4	70.2 ± 26.7	0.8

Since the pregnancy outcome is dependent on number of other factors like age, baseline FSH, indication for IVF, peak E2, total number of oocytes obtained, number of cleaved embryos and endometrial thickness on day of ET, we performed a multiple logistic regression analysis and found that after adjusting for the aforementioned variables, there was no correlation between % E2 fall and the risk of clinical pregnancy. However, the type of COH protocol used was found to be an independent variable affecting clinical pregnancy.

**Figure 1:**
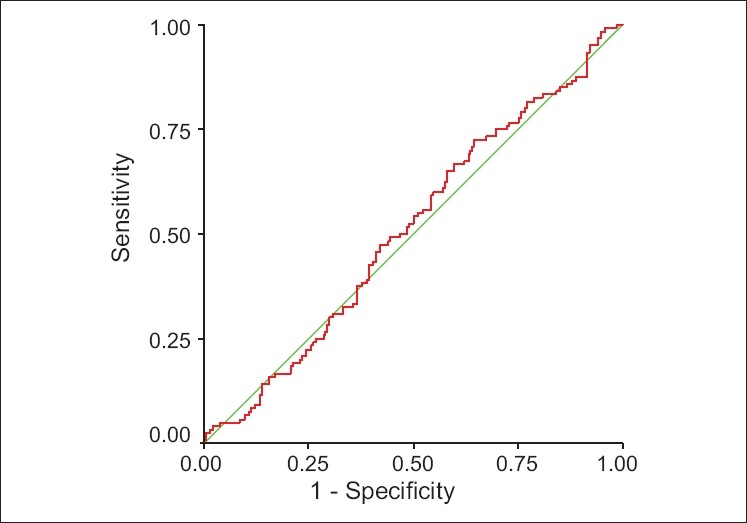
ROC curve for %EL-E2 decline

**Figure 2:**
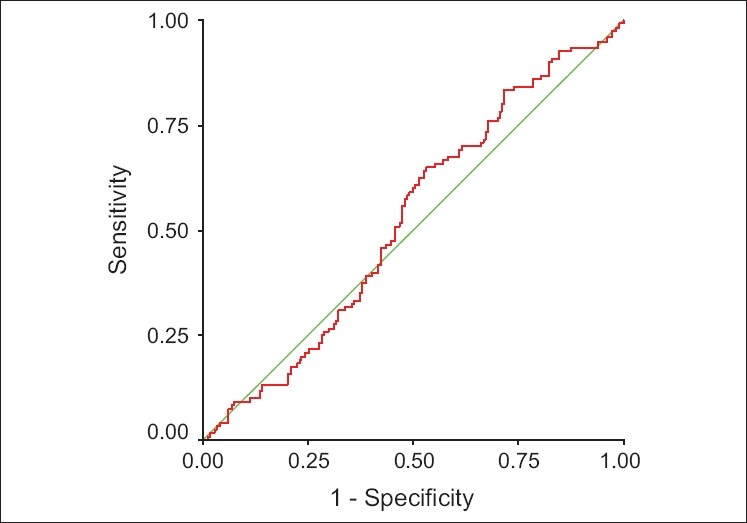
ROC curve for %ML-E2 decline

We calculated the area under the curve to assess the predictive value of %EL-E2 decline and %ML-E2 decline for discriminating between conception and nonconception cycles. The AUC suggests no relationship between %EL-E2 decline (0.51, 95% CI = 0.4-0.5), % ML-E2 decline (0.52, 95% CI = 0.4-0.5) and the clinical pregnancy rate [Figures 1 and 2].

We also analyzed whether the risk of bleeding in the first trimester among ongoing pregnancies was in some way related to the %E2 fall but failed to find any such correlation [Table 5]

**Table 5:** Effect of midluteal E2 decline on risk of first trimester bleeding

E2 decline (%)	Bleeding PV (*N* = 10)	No bleeding PV (*N* = 89)	*P* value
ML-E2 decline	64.8 ± 26.4	70.9 ± 27.1	0.5
EL-E2 decline	43.6 ± 25	38.9 ± 18	0.4

## DISCUSSION

The present study was conducted to determine the impact of %E2 fall in the early and midluteal phase on pregnancy outcome in three COH protocols.

We found that the %E2 fall in the early as well as in the midluteal phase did not have an impact on the clinical pregnancy rate as well as the first trimester miscarriage rate.

In ART cycles, the luteal phase is characterized by rapid changes in the hormonal levels. Just before the onset of the luteal phase supraphysiologic E2 concentrations are reached. This is followed by a dramatic fall in the E2 levels after oocyte aspiration. The steroidal levels fall further by the day 9 of oocyte trigger as the trophic HCG stimulus to the corpus luteum gets weaned off.[2]

Stewart *et al*.[3] were the first to identify a significant difference in serum E2 concentrations between conception and nonconception cycles in fertile women undergoing donor insemination.

Later, Sharara *et al*.[4] studied the impact of midluteal E2 fall in long luteal protocol in 106 patients undergoing ART. Luteal phase supplementation using 50-100 mg of intramuscular progesterone in oil was given to all patients. They observed that the magnitude of E2 fall as measured by the ratio of E2 on day of HCG and midluteal E2 was highly predictive of successful IVF outcome and a sharp decline in the E2 (ratio >5) resulted in a significantly lower implantation and pregnancy rates. This raised the speculation that the E2 fall would compromise the peri-implantation endometrial development.

Levi *et al*.[5] studied the luteal phase of 35 women undergoing long luteal and flare protocols and ICSI. They determined the E2 fall on days 4, 7, 9, 11 of ET. The luteal phase was supported by progesterone gel and HCG injections. They concluded that the steeper the decline in E2 levels, the lower was the chance of conception. It was speculated that insufficient exposure of the endometrium to E2 might lower or inhibit maintenance of the effects of some factors like leukemia inhibiting factor, interleukin-1, TNF-α, and interferone.

Recently, Ganesh *et al*.[6] published a study involving 268 women undergoing midluteal downregulation and supported by vaginal progesterone in the luteal phase. They found that E2 on day 7 after ET was significantly higher in the conception group as compared to the nonpregnant group. They attributed this to the corpus luteal rescue brought about by the trophoblastic HCG after implantation.

The results of these studies are refuted by the observations by Ng *et al*.[7] and several other studies and case reports listed below.

Ng *et al*.[7] studied 763 ART cycles undergoing midluteal downregulation. The luteal phase support involved HCG injections and injectable progesterone if peak E2 exceeded 18000 pmol/l. They noted that the pregnancy rate was not statistically different when the estradiol ratio was <5 or >5.

Friedler *et al*.,[8] studied the luteal phase E2 levels in 100 patients undergoing midluteal downregulation. All patients were supported by micronized progesterone in the luteal phase. No difference was observed in the mean midluteal E2 levels and %E2 decline in the conception and nonconception cycles.

Cases of successful outcomes have been reported when supplemental luteal E2 was inadvertently omitted in donor oocytes cycles.[9,10]

Women with absent or inactive ovaries, when supplemented with progesterone only, after 14 days of estrogen priming, developed normal secretory endometrial changes on immunochemistry.[11]

In a recent meta-analysis comparing the effect of the combination of E2 and progesterone verses progesterone alone for luteal phase support, it was concluded that addition of E2 to progesterone does not increase the pregnancy rate in the GnRH agonist as well as antagonist cycles.[12]

According to the Cochrane review, there was no evidence that the addition of estrogen to progesterone for luteal phase support improves ART outcomes in GnRHa cycles.[13]

In the current, study, we estimated the E2 fall immediately after OPU and again on day 7 of ET. We observed that by day 7 of ET, E2 levels fall dramatically (>75% decline in 49% patients). The decline is maximum in the long midluteal downregulation. Such dramatic fall in E2 has been reported By other authors.[4,8]

We compared the mean %EL-E2 and %ML-E2 decline in patients with preclinical, clinical abortions, and ongoing pregnancy but failed to demonstrate any significant difference. Contrary to this, Friedler *et al.* reported, that the early spontaneous abortions were higher when the %E2 decline was >98%.[8] One drawback while interpreting this data is that we did not rule out genetic causes as none of our patients opted for chromosomal analysis.

We also studied the effect of %ML - E2 decline on the risk of encountering bleeding/spotting PV in first trimester among those who crossed 12 weeks of gestation and found no correlation with same.

The follicular estradiol has a progesterone priming action. It has been demonstrated that the progesterone receptor levels are highest during the preovulatory and immediate postovulatory periods during which serum estrogen titres are highest.[14] However, in the luteal phase, progesterone has an anti-estrogen action. It inhibits estrogen in two ways: Firstly, by inhibiting synthesis of E2 receptors and secondly, by increasing the synthesis of 17 β hydroxyl dehydrogenase in the glands, which convert estradiol into estrone. Estrone has a weak affinity for estrogen receptor. Morphologic studies indicate that the estrogen and progesterone receptors fall sharply after ovulation with the estrogen receptors disappearing completely from the stroma. The predecidual cells are characterized by the absence of estrogen receptors and presence of subtype A progesterone receptors, which primarily mediate the progesterone action.[14]

Groll *et al*.[15] studied the effects of variations in serum estradiol concentrations on secretory endometrial development in experimentally induced cycles in normal women with previously proven fertility. Eighteen women underwent pituitary downregulation with leuprolide, followed by a 10-day treatment with 0.2 mg/d transdermal estradiol (E2) with subsequent allocation to one of the two 10-day estradiol regimens plus 40 mg daily intramuscular P: Supraphysiologic (0.2 mg/d transdermal E2 or 6 to 12 mg/day of vaginal micronized E2) or subphysiologic (no exogenous E2 treatment). They observed with immunohistochemistry and immunoblotting that the expression of the b3 integrin subunit and osteopontin (OPN), a biomarker for endometrial receptivity, were similar in the supraphysiologic and subphysiologic groups. Their observations suggest that, probably, the endometrial receptivity is not affected with wide variations of estradiol levels provided progesterone is supplemented.

## CONCLUSION

Our observations suggest that estradiol decline in the luteal phase does not seem to have a harmful effect on IVF outcome. Larger studies are needed to validate this data.
